# Expression and promotor hypermethylation of miR-34a in the various histological subtypes of ovarian cancer

**DOI:** 10.1186/s12885-016-2135-2

**Published:** 2016-02-15

**Authors:** Gabriel Schmid, Sara Notaro, Daniel Reimer, Samira Abdel-Azim, Michaela Duggan-Peer, Jessica Holly, Heidi Fiegl, Julia Rössler, Annemarie Wiedemair, Nicole Concin, Peter Altevogt, Christian Marth, Alain Gustave Zeimet

**Affiliations:** Department of Obstetrics and Gynaecology, Medical University of Innsbruck, Anichstrasse 35, 6020 Innsbruck, Austria; Department of Gynecology and Obstetrics, University of Brescia, Piazza Spedali Civili 1, 25123 Brescia, Italy; Skin Cancer Unit, German Cancer Research Center (DKFZ), Heidelberg, Germany; Department of Dermatology, Venereology and Allergology, University Medical Center Mannheim, Ruprecht-Karl University of Heidelberg, Theodor-Kutzer-Ufer 1-3, 68167 Mannheim, Germany

**Keywords:** miR-34a, Ovarian cancer, Epigenetics, Promotor hypermethylation, Gene-silencing

## Abstract

**Background:**

An increasing body of evidence shows that miR-34 family has tumor suppressive properties mediating apoptosis, cell cycle arrest and senescence. In ovarian cancer, miR34 family members were found to be under expressed. Particularly miR-34a has been revealed to be a direct transcriptional target of p53 which is frequently mutated in epithelial ovarian carcinomas especially in high grade serous cancer. Moreover, methylation of miR-34a CpG Islands was found to down-regulate miR-34a expression. The aim of this study was to investigate the clinical relevance of mir34a as well as its promoter methylation in a subset of 133 ovarian cancers with a special focus on the p53 mutation status, the dualistic type I and type II ovarian cancer model and the different histotypes.

**Methods:**

One hundred thirty-three epithelial ovarian cancers and 8 samples of healthy ovarian surface epithelium were retrospectively analysed for miR-34a expression with quantitative real-time reverse transcription PCR (qRT-PCR). Gene-specific DNA methylation was evaluated with MethyLight technique.

**Results:**

Significantly lower miR-34a expression was found in ovarian cancers than in healthy ovarian epithelium (*p =* 0.002). The expression of miR-34a was found lower in type II than in type I cancers (*p =* 0.037), in p53 mutated as compared to p53 wild type cancers (*p =* 0.003) and in high grade compared to in low grade cancers (*p =* 0.028). In multivariate COX regression model low expressing miR-34a cancers exhibited a reduced PFS (*p =* 0.039) and OS (*p =* 0.018). In serous cancers low miR-34a levels showed a worse OS confirmed also in multivariate analysis (*p =* 0.022). miR-34a promoter methylation was found higher in type II cancers than in type I (*p =* 0.006). mir34a expression and promoter methylation showed an inverse correlation in cancer samples (*p =* 0.05).

**Conclusion:**

We demonstrated a clinical independent role of miR-34a in epithelial ovarian cancers. Moreover, we corroborated the correlation between miR-34a expression and its promoter methylation in a large set of ovarian cancers. The inverse association between miR-34a expression and grading, p53 mutation status and dualistic tumor type classification, together with its prognostic relevance may underline the tumor-suppressive character of miR-34a in ovarian cancer.

## Background

In epithelial ovarian carcinomas p*53* is mutated in more than 50 % of cases; especially high-grade serous cancer is one of the most impressive p53 mutation-driven malignancies with a mutation frequency of more than 95 % [1]. Based on clinical behaviour, histological characteristics as well as genetic features Kurman et al. [2] proposed a dualistic model to group epithelial ovarian cancer. Type I cancers arise from borderline tumors and are often limited to the ovaries at time of diagnosis. They frequently show mutations of RAS, BRAF and PTEN. In contrast, type II carcinomas are frequently deficient in the p53 pathway, are more aggressive and thus often widely spread through the peritoneal cavity at time of diagnosis [1, 3]. Although first-line treatment is successful in most cases, 5-year overall survival (OS) is still poor which is mainly due to the very high incidence of early recurrence and the development of platinum resistance during the course of the disease [4].

Micro RNA (miRs) are small non-coding RNAs that appear to play an important role in cancer development and their dysregulation is a ubiquitous feature of malignancies [5]. The miR-34 family is one of the most prominent miR groups, known to be crucially involved in carcinogenesis and tumor progression. Members of the miR-34 family can act either as oncogenes or as tumor-suppressors by regulating the cell cycle, proliferation, apoptosis, invasion and metastasis [6]. The tumor-suppressor and transcription factor p53 has been shown to directly transactivate genes of the miR-34 family [7, 8].

Accumulating evidence suggests that many malignancies follow a stem cell model, where a subpopulation of tumor cells with stem cell properties drives tumor growth, invasion and metastasis [9]. Because of their relative resistance to conventional therapies, such as chemotherapy or radiation, these cells may be responsible for treatment resistance and recurrence. A recent work by Liu et al. [10] showed that miR-34a inhibits expression of the adhesion molecule CD44 in prostate cancer stem cells, and is able to block tumor growth and metastasis formation in a xenograft model. Furthermore, these authors also revealed that miR-34a reduces the expression of other molecules crucially involved in the regulation of stem cell pathways including cyclin D1, cyclin-dependent kinase-4 and −6, c-myc as well as NOTCH. Altogether, these findings prompted Max Wicha to highlight the striking role of miR-34a in the biology of cancer stem cells in one of his renowned editorial entitled “Stemming a tumor with a little miR” [11].

The control system of miR-34a seems to be complex, in fact p53 has been revealed to up-regulate the expression of miR-34a via direct promotor transactivation [8, 12] but also miR-34a appeared to be regulated by epigenetic regulation via its specific promoter methylation [13]. The latter Moreover, proved to be decisive in regulating this miR-34a-E2F3a axis [12].

All this motivated us to investigate the clinical relevance of miR-34a as well as its promotor methylation status in a training set of 133 ovarian cancer patients. In this context particular attention was focused on differences between type I and type II cancers, p53 mutation-driven cancers and the various histological subtypes.

## Methods

We investigated tissue samples from 133 patients with invasive, epithelial ovarian cancer. These samples were collected at the Department of Obstetrics and Gynecology at the Medical University of Innsbruck during primary surgery. Tumor specimen were obtained immediately after surgery and brought to our pathology laboratory, where the tissue was pulverized under cooling with liquid nitrogen and stored at −70 °C. Tumors with borderline malignancy were excluded. Normal ovarian surface epithelial tissue samples obtained from ovaries removed for other than inflammatory or tumoral conditions served as controls (*n =* 8).

In the present training set of patients the median age at diagnosis was 62.3 years (Range: 51–71.9). Clinico-pathological characteristics of the investigated patients are summarized in Table 1.

**Table 1:** Clinico-pathological characteristics of the investigated cohort (*n =* 130)

		miR-34a expression^d^							miR-34a promoter methylation			
		n. (%)		Missing	Median	*p*-value^b^	n. (%)		Missing	Negative	Positive	*p*-value^c^
Presence of disease	healthy samples	8	5,7 %	0	1,39		8	5,7 %	8	-	-	
	cancers	133	94,3 %	38	0,39	**0.002** ^**f**^	133	94,3 %	37	61	34	-
Age	<62.3 years^a^	66	49,6 %	16	0,44		66	49,6 %	19	30	17	
	>62.3 years	67	50,4 %	22	0,33	0.136	67	50,4 %	18	31	17	0.939
Histotype	serous	67	50,4 %	22	0,33		67	50,4 %	14	32	21	
	endometrioid	24	18,0 %	6	0,5		24	18,0 %	6	12	5	
	mucinous	37	27,8 %	10	0,4		37	27,8 %	15	15	7	
	clear cell	5	3,8 %	0	1,07	0.215	5	3,8 %	2	2	1	0.799
FIGO stage	early stages (I-II)	31	23,3 %	9	0,4		31	23,3 %	10	15	6	
	late stages (III-IV)	102	76,7 %	29	0,36	0.192	102	76,7 %	27	46	28	0.434
Grading	1	4	3,0 %	1	1,43		4	3,0 %	0	4	0	
	2	74	55,6 %	23	0,44		74	55,6 %	21	37	15	
	3	55	41,4 %	14	0,33	**0.028** ^**e**^	55	41,4 %	16	20	19	0,046^e^
Residual disease	RD = 0	49	37,1 %	9	0,42		49	37,1 %	19	23	7	
	RD > 0	83	62,9 %	29	0,36	0.491	83	62,9 %	17	38	27	0,085
Type	type 1	91	68,4 %	26	0,43		91	68,4 %	28	45	17	
	type 2	42	31,6 %	12	0,28	**0.037** ^**e**^	42	31,6 %	9	16	17	**0,020** ^**e**^
Serous	LGSC	13	29,5 %	6	0,77		13	29,5 %	2	8	3	
	HGSC	31	70,5 %	12	0,21	**0.004** ^**f**^	31	70,5 %	7	12	12	0,207
p53 mutation	no	30	33,0 %	12	0,7		30	33,0 %	6	18	6	
	yes	61	67,0 %	19	0,33	**0.003** ^**f**^	61	67,0 %	18	22	20	0,07

^a^Median value in cancer cohort. ^b^Mann–Whitney test or Kruskal-Wallis test ^c^chi-square test. ^d^Arbitrary units normalized to TBP. ^e^Significant at the 0.05 level (2-tailed). ^f^Significant at the 0.01 level (2-tailed)

### RNA extraction, reverse transcription and real-time PCR analysis

RNA from fresh pulverized, quick-frozen specimens was extracted as previously described [14]. A TaqMan microRNA assay specific for miR-34a (Assay ID 000426) was used to detect and quantify mature miR-34a. miRNA expression was normalized to RNU6B (Assay ID 001093) using the 2-ΔΔCt method. The assays were performed in accordance with manufacturer’s instructions (Applied Biosystems, Carlsbad, USA) using an ABI Prism 7900 Detection System. PCR assays were conducted in triplicate and the mean value was used for calculation. Values were expressed with TBP: TATA box-binding protein.

### DNA isolation and methylation analyses

Genomic DNA from pulverized, quick-frozen specimens was isolated using the DNeasy tissue kit (Qiagen, Hilden, Germany). Bisulfite modification was performed with the EZ DNA Methylation-Gold Kit (Zymo Research, Orange, CA, USA) according to the manufacturer’s instructions. MethyLight analysis was done as described previously [12]. Primers and probes for miRNA34a were determined with the computer program Primer Express, version 2.0.0 (Applied Biosystems, Foster City, CA, USA), to produce a 111-base-pair PCR amplicon (nucleotide positions 33.932–34.043 as defined by GenBank accession number EF570049; −192 nucleotides to −81 nucleotides upstream from exon 1). Genomic DNA not treated with bisulfite (unmodified) was not amplified with the primers (data not shown).

Primer sequences were: miR-34a Forward 5′-TCCTTCCTACTCGTACCACCAAA-3′, miR-34a reverse 5′-AGGTGGAGGAGATGTCGTTGTT-3′, miR-34a Taq Man probe 5′FAM-CGTCTCTCCAACCCGAAATCCGAAAAA-3′-BHQ1. CpG islands in the analyzed genes were identified using a CpG island searcher (http://www.cpgislands.com) which screens for CpG islands that meet the criteria and algorithm described by Takai and Jones [15]. Values were expressed as PMR value: percentage of fully methylated reference;

### Statistical analysis

Data were expressed as median values and interquartile ranges. Differences between two and more than two quantitative variables were evaluated using respectively Mann Whitney and Kruskal-Wallis Test. Correlation analyses were calculated using Spearman rank-correlation test. Survival analysis for PFS and OS was performed with the Kaplan-Meier method and statistical differences were evaluated with log-rank test. Multivariate analysis (Cox-regression) was performed to determine the impact of clinical and pathologic factors on survival outcomes. SPSS, version 22 for Windows, was used for all analyses.

## Results

### miR-34a expression and its promotor methylation in cancer and normal ovarian tissue specimens

From the 133 patients miR-34a expression (miR-34a^(EXP)^) was available in 95 patients and promotor methylation of miR-34a (miR-34a^(MET)^) in 96 patients. For 58 patients data were available for both variables. The median expression level of miR-34a was significantly lower in the cancer specimen than in the healthy control tissue (*p =* 0.002, Table 1). No promoter methylation could be measured in the control tissues. In cancers 61 patients (63.5 %) methylation miR-34a promoter was totally absent presented absent methylation of and the remaining 35 (36.5 %) had various levels of methylation (PMR values) ranging from 0.5 to 30.42.

### According to the clinico-pathological characteristics

**Fig. 1:**
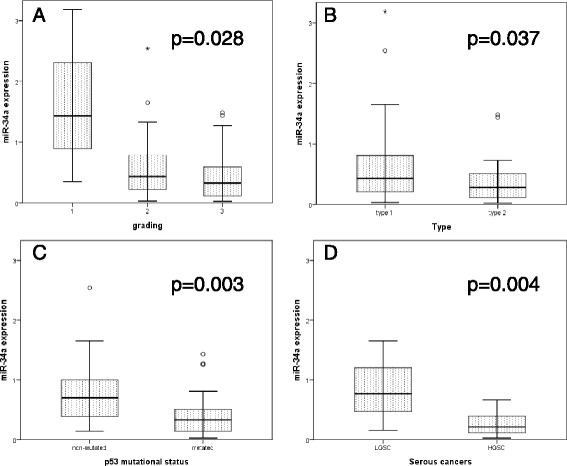
miR-34a expression in different clinico-pathologic characteristics. **a** in grade 1, 2 and 3 ovarian cancers. **b** in type I and type II tumors (according to the dualistic classification of Kurman et al.). **c** according to p53 mutational status. **d**) in low grade serous carcinomas (LGSC) and high grade serous carcinomas (HGSC). P-value is calculated with non-parametric tests (Mann Whitney for between two variable and Kruskal Wallis test between three or more variables. Y-axis represents the value of miR-34a expression as arbitrary unit normalized to TBP

Regarding histo-morphological grading Fig. 1a depicts that miR-34a^(EXP)^ was revealed to be higher in grade 1 as compared to grade 2 and 3 (*p =* 0.028, Table 1). Cancers positive for miR-34a promoter methylation were significantly more represented (*p =* 0.046) in grade 2 and 3 than in grade 1 (Table 1).

According to the dualistic type I and II model proposed by Kurman et al. [2] miR-34a^(EXP)^ was significant lower in type II than in type I cancers (*p =* 0.037) and miR-34a^(MET)^ was significantly higher in type II than in type I (*p =* 0.020); (Table 1, Fig. 1b). In 36.4 % of the whole cohort of patients p53 mutation was revealed. p53 mutated cancers exhibited significantly lower miR-34a^(EXP)^ than did p53 wild-type cancers (*p =* 0.003, Fig. 1c). However, no differences between p53 mutated and wild-type cancers could be revealed with regard to miR-34a specific promotor methylation (Table 1). When we separately analysed the serous histological subtype we revealed that high grade cancers showed a significant lower miR-34a^(EXP)^ than the low grade serous cancers (*p =* 0.004); (Table 1, Fig. 1d).

**Fig. 2:**
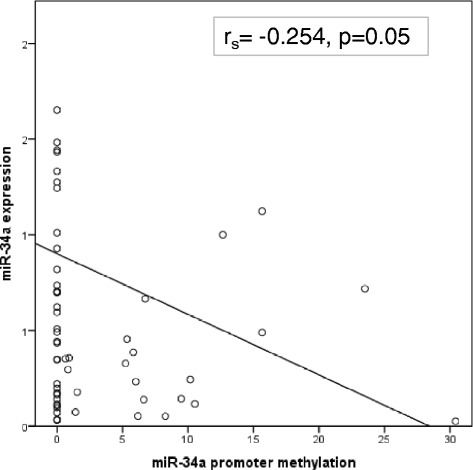
Correlation between miR-34a expression and miR-34a methylation. Graphic representation of the inverse linear correlation between miR-34a expression and its promoter methylation. Y-axis represent miR-34a expression as arbitrary units normalized to TBP and X-axis represent methylation of miR-34a as PMR value (percentage of methylated reference)

In accordance with the assumption that full methylation of CpG islands in a promotor leads to gene silencing, expression of miR-34a (miR-34a^(EXP)^) and methylation status of its promotor (miR-34a^(MET)^) showed negative correlation (r_s_ = −0.254, *p =* 0.05); Fig. 2.

### Survival analysis

As no data at all are available on clinical relevance of miR-34a expression, we calculated an optimal cut-off for both by stratifying patients of this training set into 2 groups according to their miR-34a expression, using various cut-off points set arbitrarily between the 20th and 80th percentile. Survival curves were calculated for each of these cut-offs, and p values were calculated. The optimal cut-off point for miR-34a expression with the highest level of significance was the 20th percentile which was used as discriminator for miR-34a positive and negative expression status. Regarding miR-34a methylation we used as threshold the presence or the absence of methylation, however, we were not able to find significant survival differences in our cohort with regard to miR-34a promoter methylation.

**Fig. 3:**
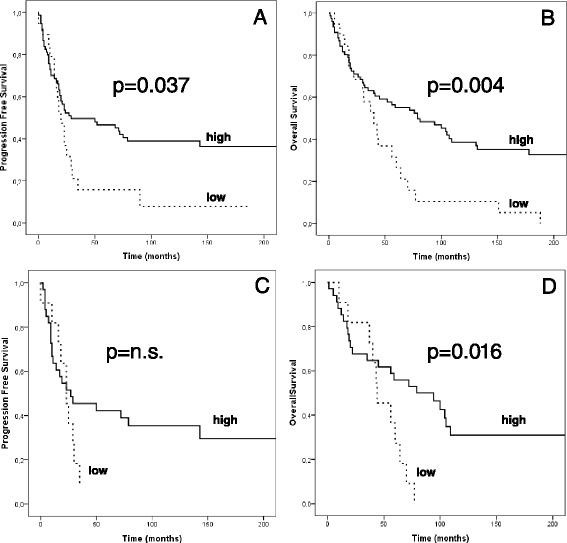
Univariate survival analysis based on miR-34a expression. **a**-**b** PFS and OS in the entire cohort according to high and low miR-34a expression level. **c**-**d** PFS and OS in serous cancers according to high and low miR-34a expression level. Cut off used for miR-34a expression: 20th percentile of the entire cancer cohort (see results section for details). P-value calculated with log-rank test

Univariate survival analysis for miR-34a^(EXP)^ at the 20th-percentile for PFS and OS shows that tumor specimens with low miR-34a expression have significant worse PFS (*p =* 0.037) and OS (*p =* 0.004) (Fig. 3a and b).

In the multivariate Cox regression model, including the variables FIGO stage, grading, residual disease and histological subtype, miR-34a^(EXP)^ retained independent prognostic power both for PFS (HR 0.549 (95 % CI 0.310–0.969), *p =* 0.039) and OS (HR 0.509 (95 % CI 0.293–0.883), *p =* 0.016) (Table 2).

**Table 2:** Multivariate cox regression survival analysis

		PFS			OS		
		HR	CI 95 %	*p* value	HR	CI 95 %	*p* value
*miR-34a* expression^c^	high	0.549	[0.31–0.969]	0.039^a^	0.509	[0.293–0.883]	0.016^a^
	low	1					
FIGO stage	late	1.646	[0.7–3.871]	0.253	2.058	[0.895–4.727]	0.089
	early	1					
Age	>62.3	1.69	[1.017–2.813]	0.043^a^	2.565	[1.571–4.189]	<0.0001^b^
	<62.3	1					
Residual disease	RD > 0	1.97	[1.057–3.674]	0.033^a^	2.565	[1.571–4.189]	0.001^b^
	RD = 0	1					
Hystology	^d^	0.998	[0.773–1.288]	0.987	1.132	[0.886–1.446]	0.321
		Serous cancers					
		PFS			OS		
		HR	CI 95 %	*p* value	HR	CI 95 %	*p* value
*miR-34a* expression^c^	high	0.509	[0.227–1.143]	0.102	0.389	[0.173–0.876]	0.022^a^
	low	1					
FIGO stage	late	2.866	[0.77–10.662]	0.043	2.858	[0.748–10.915]	0.125
	early	1					
Age	>62.3	2.304	[1.082–4.905]	0.032	2.925	[1.386–6.174]	0.005
	<62.3	1					
Residual disease	RD > 0	1.194	[0.456–3.128]	0.718	1.080	[0.397–2.938]	0.879
	RD = 0	1					

^a^significant at the 0.05 level (2-tailed). ^b^significant at the 0.01 level (2-tailed). ^c^arbitrary units normalized to TBP, cut off = 20th centile (0.14)

^d^serous VS endometrioid VS mucinous VS clear cell

Survival analysis was performed separately for the various histological subtypes excluding the clear cell cancers which were underrepresented. In serous cancers the univariate approach showed a worse OS in case of low miR-34a values (*p =* 0.016) but was not able to demonstrate a significance difference in PFS (Fig. 3b–c). This worse OS was confirmed in the multivariate setting (HR 0.394 (95 % CI 0.178–0.873) *p =* 0.022, Table 2. In mucinous cancers low expression of miR-34a in univariate analysis showed a borderline worse PFS (*p =* 0.052) which could not be confirmed in multivariate analysis.

In none of the analysed histological subtypes methylation of the miR-34a promoter was found to be of prognostic relevance.

## Discussion

Gene-silencing through promotor methylation is a unique and reversible mechanism in the context of epigenetic regulation of the genome in general. Accordingly, in many human cancers promotor hypermethylation of tumor-suppressor genes is detected. MiRNAs represent another epigenetic machinery for controlling definitive gene expression in so far as translation of transcripted mRNA is specifically abrogated via mRNA degradation after selective binding by miRNAs.

We investigated the clinical impact of miR-34a as well as the methylation status of its promotor in ovarian cancer. The degree of promotor hypermethylation was significantly inversely associated with the expression of miR-34a although the association found was not strong. The low level of significance is probably due to the low number of cancer with detectable methylation. Significant inverse associations between promotor methylation and the respective gene expression are not always given especially in clinical data sets, as expression of genes often seems to be only moderately affected by hypermethylation of the respective promotor. However, our findings tempted us to speculate that in ovarian cancers miR-34a-driven epigenetic regulation of gene translation is partially governed by a second epigenetic mechanism, namely DNA methylation. This is an excellent example that shows how epigenetic regulation itself is controlled by epigenetics.

Univariate and multivariate survival analyses performed in the whole cohort of patients revealed that tumoral miR-34a expression below the 20 % percentile was associated with poor PFS and OS.

This is in agreement with the recently reported finding that miR-34a is also a crucial suppressor of the cell cycle promoting factor E2F3a, which was revealed to be a very important regulator in EGFR-driven growth signaling in ovarian cancer [16]. The epigenetic control by promotor methylation of the miR-34a gene, has been shown to increase E2F3a expression in vivo and highlights miR-34a as a substantial regulator in ovarian cancer biology [14]. In this context, it has been previously shown that miR-34a expression is directly inducible by p53 and putatively involved in G1 arrest and apoptosis. Accordingly, it was found that ovarian cancers with mutations leading to p53 inactivation, were associated with significantly lower levels of miR-34a^(EXP)^ and higher levels of E2F3a [12].

Furthermore, it has been revealed that only an intact p53/miR-34a axis is able to suppress L1CAM [13]. Expression of L1CAM has been found to correlate with poor prognosis and metastasis in ovarian and endometrial carcinomas [17, 18]. L1CAM cleavage and its concomitant release of the soluble molecule have been shown to promote migration, invasion and protection from apoptosis of cancer cells. After all, the miR-34a regulated L1CAM expression contributes to the invasive and metastatic phenotype of serous ovarian carcinoma [19].

P53 wild-type directly transactivates miR-34a, and may execute, at least partially, its function as a tumor-suppressor via up-regulation of miR-34a. This is underscored by the biologically relevant connection between the expression of miR-34a and the p53 functional status in our cohort of patients. However, it appears that p53 is not the only determining factor of miR-34a expression and miR-34a promotor methylation status plays a significant role in miR-34a expression in ovarian cancer cells.

Even though the p53 mutation-driven type II ovarian cancers exhibit lower miR-34a expression and higher methylation of the miR-34a promotor than do type I cancers, no association between the methylation status of miR-34a and p53 function could be revealed.

It is well known that restoration of functional p53 is an attractive approach for gene therapy due to the large number of p53 transcription targets, including miR-34a [20]. However, first attempts at p53 gene therapy in ovarian cancer patients failed to prove their therapeutic efficiency in a randomized clinical phase 3 trial [21]. Possibly, miR-34a could be more attractive than p53 due to its smaller molecular size, making it more prone for viral or non-viral transfection.

Wei et al. [22] investigated the clinical importance of miR-34a in gastric cancer specimens and revealed that miR-34a is a valuable predictor of favorable prognosis. miR-34a was shown in vitro to act as a tumor suppressor inhibiting gastric cancer cell proliferation and invasion via downregulation of MET. This also points out that miR-34a has different targets to execute its function as tumor suppressor.

In ovarian cancer another target of miR-34a is AXL, a tyrosine kinase receptor with oncogenic properties. Recently Li et al. [23] found in vitro that miR-34a suppresses ovarian cancer proliferation and motility by targeting AXL.

A work from 2011 Vogt et al. [24] showed that miR-34a and miR-34b/c are inactivated by CpG methylation. They investigated promoter methylation in several tumor types including ovarian cancers. From 13 ovarian cancer samples analysed from ovarian cancer 8 (61.54 %) showed methylation of the CpG).

## Conclusion

The inverse correlations between miR-34a expression and grading, p53 mutation status and dualistic tumor type classification, together with its prognostic relevance shown herein in multivariate survival analysis may underline the tumor-suppressive property of miR-34a in ovarian cancer. Altogether, these findings underscore the relevance of miR-34a in ovarian cancer cell biology with special regard to its inhibitory effect on proliferation and invasion.

The clinical impact shown in this training set needs to be confirmed in a larger study population with regard to the various histological and molecular-biological subtypes. Beyond its prognostic relevance miR-34a could in fact open new avenues in the development of innovative treatment approaches modulating the expression of a number of molecules decisively involved in the progression of ovarian cancer.

## Ethics

The study was conducted in accordance with the principles of the Helsinki Declaration after approval by the local ethics committee (Ethikkommission der Medizinischen Universität Innsbruck, Innrain 43, A-6020 Innsbruck) for both the case and control participants. Clinical, pathological and follow-up data were stored in a database in accordance with hospital privacy rules. Tumor samples and clinical data as well as control samples were collected after obtaining written informed consent.

## Abbreviations

OS: overall survival
PFS: progression free survival
CI: confidence interval
HR: hazards ratio
MiR: micro RNA
